# Circulating soluble urokinase plasminogen activator receptor is stably elevated during the first week of treatment in the intensive care unit and predicts mortality in critically ill patients

**DOI:** 10.1186/cc10037

**Published:** 2011-02-16

**Authors:** Alexander Koch, Sebastian Voigt, Carsten Kruschinski, Edouard Sanson, Hanna Dückers, Andreas Horn, Eray Yagmur, Henning Zimmermann, Christian Trautwein, Frank Tacke

**Affiliations:** 1 Department of Medicine III, RWTH-University Hospital Aachen, Pauwelsstrasse 30, 52074 Aachen, Germany; 2 Institute of Family Medicine, Hannover Medical School, Carl-Neuberg-Strasse 1, 30625 Hannover, Germany; 3 Medical Laboratory Dr. Stein, Robert-Koch-Strasse 2, 42549 Velbert, Germany

## Abstract

**Introduction:**

suPAR is the soluble form of the urokinase plasminogen activator receptor (uPAR), which is expressed in various immunologically active cells. High suPAR serum concentrations are suggested to reflect the activation of the immune system in circumstances of inflammation and infection, and have been associated with increased mortality in different populations of non-intensive care patients. In this study we sequentially analyzed suPAR serum concentrations within the first week of intensive care in a large cohort of well characterized intensive care unit (ICU) patients, in order to investigate potential regulatory mechanisms and evaluate the prognostic significance in critically ill patients.

**Methods:**

A total of 273 patients (197 with sepsis, 76 without sepsis) were studied prospectively upon admission to the medical intensive care unit (ICU), on Day 3 and Day 7, and compared to 43 healthy controls. Clinical data, various laboratory parameters as well as investigational inflammatory cytokine profiles were assessed. Patients were followed for approximately one year.

**Results:**

Upon admission to the ICU suPAR serum concentrations were elevated in critically ill patients as compared with healthy controls. In sepsis patients suPAR levels were higher than in non-sepsis patients (with or without systemic inflammatory response syndrome (SIRS)). During the first week after admission to the ICU serum suPAR concentrations remained stably elevated. suPAR serum concentrations measured upon admission were closely and independently correlated to various laboratory parameters, specifically biomarkers of inflammation (tumor necrosis factor (TNF), C-reactive protein (CRP)), hepatic and renal dysfunction. High suPAR levels at admission and at Day 3 were a strong independent predictor for both ICU and long-term mortality in critically ill patients.

**Conclusions:**

In sepsis and non-sepsis patients suPAR serum concentrations are increased upon admission to the ICU, likely reflecting the activation state of the immune system, and remain stably elevated in the initial course of treatment. Low suPAR levels are a positive predictor of ICU- and overall survival in critically ill patients, including sepsis and non-sepsis patients. Aside from its value as a promising new prognostic biomarker, both experimental and clinical studies are required in order to understand the specific effects and regulatory mechanisms of suPAR in SIRS and sepsis, and may reveal new therapeutic options.

## Introduction

The urokinase plasminogen activator receptor (uPAR) is expressed on different cell types including neutrophils, lymphocytes, monocytes, macrophages, certain cancer cells and vascular endothelial cells. uPAR and its ligand urokinase plasminogen activator (uPA) are participants in numerous immunologic functions including migration, adhesion, angiogenesis, fibrinolysis and cell proliferation and have been found to promote tissue invasion in malignant diseases by converting plasminogen into plasmin, resulting in degradation of extracellular matrix [1-4]. Migration of inflammatory cells from the blood stream into tissues is also an essential component of inflammation and immune response against infection, in which the uPAR/uPA system is directly involved [5].

Through inflammatory stimulation uPAR is cleaved from the cell surface by proteases to the soluble form of the receptor, suPAR, which has been detected in blood, urine and cerebro-spinal fluid [6-9]. Increased activation of the immune system caused by different types of infections or various solid tumours, results in increased suPAR concentrations in body fluids. Thereby serum suPAR levels are believed to mirror the degree of immunoactivation. Moreover, high suPAR serum concentrations have been found to predict mortality in patients with active tuberculosis and in healthy subjects [10]. In a recent study, high suPAR serum concentrations have been shown to indicate a poor outcome in patients with systemic inflammatory response syndrome (SIRS) admitted to an emergency department and to a department of infectious diseases without an intensive-care environment [11]. Yet, these findings have been regarded as possibly applicable only to patients with community-acquired infections, which did not require intensive-treatment, and the validity for critically ill patients was questioned.

The present study was conducted with a large cohort of well characterized critically ill patients in a medical ICU to provide information on suPAR serum concentrations in different circumstances of critical disease, to identify potential regulatory mechanisms of suPAR by correlations with a wide number of markers of inflammation, organ dysfunction and metabolism and to elucidate the prognostic impact of suPAR in critically ill patients.

## Materials and methods

### Study design and patient characteristics

The study protocol was approved by the local ethics committee and conducted in accordance with the ethical standards laid down in the 1964 Declaration of Helsinki (ethics committee of the University Hospital Aachen, RWTH-University, Aachen, Germany, reference number EK 150/06). We enrolled 273 patients (172 male, 101 female with a median age of 64 years; range 18 to 90 years) in our study who were admitted to the General Internal Medicine ICU at the RWTH-University Hospital Aachen, Germany (Table 1). Written informed consent was obtained from the patient, his or her spouse or the appointed legal guardian. Not included in this study were patients who were expected to have a short-term (< 72 h) intensive care treatment due to post-interventional observation or acute intoxication [12]. The medium length of stay at the ICU was 9 days (range 1 to 137 days) and medium length of stay in the hospital was 27 days (range 2 to 151 days).

**Table 1:** Baseline patient characteristics and suPAR serum concentrations

Parameter	All patients	Sepsis	Non-sepsis
Number	273	197	76
Sex (male/female)	172/101	128/69	44/32
Age median (range) (years)	64 (18 to 90)	65 (20 to 90)	60 (18 to 85)
APACHE-II score median (range)	17 (0 to 40)	18 (0 to 40)	15 (0 to 31)
SOFA score median (range)	11 (0 to 17)	11 (2 to 17)	7 (0 to 16)
SAPS2 score median (range)	44 (0 to 80)	44.5 (0 to 79)	41.5 (13 to 80)
ICU days median (range)	9 (0 to 137)	12 ** (0 to 137)	6 ** (1 to 45)
Hospital days median (range)	27 (2 to 151)	30 ** (2 to 151)	14 ** (2 to 85)
Death during ICU n (%)	75 (32.8%)	60 (35.9%)	15 (24.2%)
Death during follow-up n (%)	111 (51.9%)	83 (53.5%)	28 (47.5%)
Mechanical ventilation n (%)	194 (73.2%)	144 (75%)	50 (68.5%)
Ventilation time median (range) (h)	126 (0 to 2,966)	180 * (0 to 2,966)	48.5 * (0 to 986)
pre-existing diabetes n (%)	87 (32.7%)	59 (30.7%)	28 (37.8%)
BMI median (range) (m^2^/kg)	25.8 (14.0 to 66.7)	25.9 (14.0 to 66.7)	25.8 (15.9 to 53.3)
suPAR Day 1 median (range) (ng/mL)	9.80 (0 to 20)	11.05 ** (1.87 to 20)	7.62 ** (0 to 20)
suPAR Day 3 median (range) (ng/mL)	10.83 (2.33 to 20)	12.11 * (2.59 to 20)	8.47 * (2.33 to 20)
suPAR Day 7 median (range) (ng/mL)	11.90 (3.67 to 20)	12.27 (3.94 to 20)	9.73 (3.67 to 20)

APACHE, Acute Physiology and Chronic Health Evaluation; SAPS, simplified acute physiology score; SOFA, sequential organ failure assessment.

Significant differences between sepsis and non-sepsis patients are marked by *(*P *< 0.05) or **(*P *< 0.001).

Patient data, clinical information and blood samples were collected prospectively. The clinical course of patients was observed in a follow-up period by directly contacting the patients, the patients' relatives or their primary care physician. Patients who met the criteria proposed by the American College of Chest Physicians and the Society of Critical Care Medicine Consensus Conference Committee for severe sepsis and septic shock were categorized as sepsis patients, the others as non-sepsis patients [13].

As a control population we analyzed 43 healthy blood donors (28 male, 15 female; median age 53, range 24 to 68 years) with normal values for blood counts, C-reactive protein and liver enzymes.

### Characteristics of sepsis and non-sepsis patients

Among the 273 critically ill patients enrolled in this study, 197 patients conformed to the criteria of bacterial sepsis (Table 1). Pneumonia was identified in the majority of sepsis patients as the origin of infection (Table 2). Non-sepsis patients were admitted to the ICU mainly due to cardiopulmonary diseases (myocardial infarction, pulmonary embolism, and cardiac pulmonary edema), decompensated liver cirrhosis or other critical conditions and did not differ in age or sex from sepsis patients. Sepsis patients were more often in need of mechanical ventilation in longer terms as compared to the non-sepsis patients' cohort (Table 1). In sepsis patients significantly higher levels of routinely used biomarkers of inflammation (that is, C-reactive protein, procalcitonin, white blood cell count) were found (Table 1, and data not shown). Both groups did not differ in Acute Physiology and Chronic Health Evaluation (APACHE) II, Sequential Organ Failure Assessment (SOFA) and Simplified Acute Physiology Score (SAPS)2 score, vasopressor demand, or laboratory parameters indicating liver or renal dysfunction (data not shown).

**Table 2:** Disease etiology of the study population

		Sepsis	Non-sepsis
		*n *= 197	*n *= 76
**Etiology of sepsis critical illness**			
Site of infection	n (%)		
Pulmonary		116 (59%)	
non-pulmonary		81 (41%)	
**Etiology of non-sepsis critical illness**			
	n (%)		
decompensated liver cirrhosis			19 (25%)
non-sepsis other			57 (75%)

### suPAR measurements

Blood samples were collected upon admission to the ICU (prior to therapeutic interventions) as well as in the morning of Day 3 and Day 7 after admission. After centrifugation at 2,000 g at 4°C for 10 minutes, serum and plasma aliquots of 1 mL were frozen immediately at -80°C. suPAR serum concentrations were analysed using a commercial enzyme immunoassay (ViroGates, Birkeroed, Denmark). Interleukin-6, Interleukin-10, tumour necrosis factor alpha (TNF-α) (all Siemens Healthcare, Erlangen, Germany), and procalcitonin (Kryptor, B.R.A.H.M.S. Diagnostica, Henningsdorf, Germany) were measured by commercial chemiluminescence assays, following the manufacturers' instructions.

### Statistical analysis

Data are given as median and range due to the skewed distribution of most of the parameters. Differences between two groups were assessed by Mann-Whitney-*U*-test and multiple comparisons between more than two groups have been conducted by Kruskal-Wallis-ANOVA and Mann-Whitney-*U*-test for *post hoc *analysis. Box plot graphics illustrate comparisons between subgroups and they display a statistical summary of the median, quartiles, range and extreme values. The whiskers extend from the minimum to the maximum value excluding outside and far out values which are displayed as separate points. An outside value (indicated by an open circle) was defined as a value that is smaller than the lower quartile minus 1.5-times the interquartile range, or larger than the upper quartile plus 1.5-times the interquartile range. A far out value was defined as a value that is smaller than the lower quartile minus three times the interquartile range, or larger than the upper quartile plus three times the interquartile range [14]. All values, including "outliers", have been included for statistical analyses. Correlations between variables have been analysed using the Spearman correlation tests, where values of *P *< 0.05 were considered statistically significant. All single parameters that correlated significantly with suPAR levels at admission were included in a multivariate linear regression analysis with suPAR as the dependent variable to identify independent (meaningful) predictors of elevated suPAR. The prognostic value of the variables was tested by univariate and multivariate analysis in the Cox regression model. Kaplan Meier curves were plotted to display the impact on survival [15]. Receiver operating characteristic (ROC) curve analysis and the derived area under the curve (AUC) statistic provide a global and standardized appreciation of the accuracy of a marker or a composite score for predicting an event. ROC curves were generated by plotting sensitivity against 1-specificity [16]. All statistical analyses were performed with SPSS version 12.0 (SPSS, Chicago, IL, USA).

## Results

### suPAR serum concentrations upon admission to the ICU are elevated in critically ill patients as compared with healthy controls and are higher in sepsis than in non-sepsis patients

**Figure 1:**
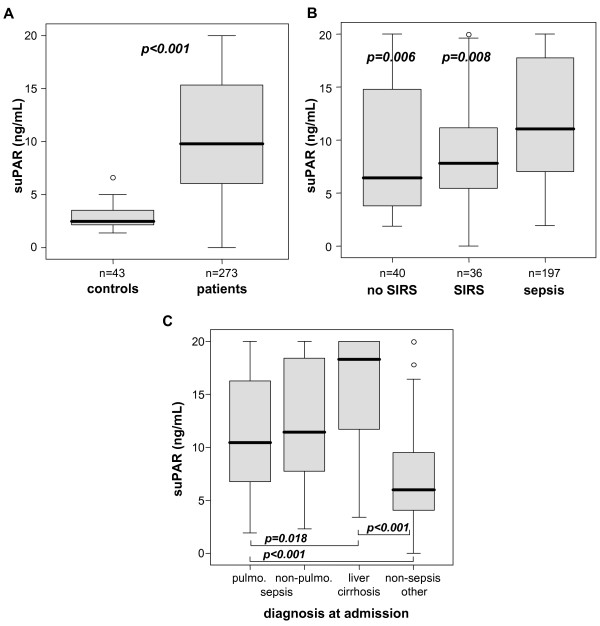
**Serum suPAR concentrations in critically ill patients with different disease etiologies at ICU admission**. **(A) **At admission to the Medical ICU, serum suPAR levels were significantly (*P *< 0.001, U-test) elevated in critically ill patients (n = 273) as compared to healthy controls (n = 43). **(B) **In comparison to ICU patients without SIRS or with SIRS, septic patients had higher suPAR serum concentrations (U-tests to sepsis group, *P*-values given in figure). **(C) **suPAR serum concentrations did not differ in patients with either pulmonary or non-pulmonary origin of sepsis and are highest in patients with decompensated liver cirrhosis.

To examine the significance of suPAR measurements at admission and during the clinical course in a medical intensive care environment we analyzed blood samples of critically ill patients at admission (= before therapeutic intervention), on Day 3 and on Day 7 (Table 1). As demonstrated in Figure 1a critical care patients had significantly higher suPAR serum concentrations as compared with healthy controls (median 2.44 ng/mL in controls versus 9.80 ng/mL in ICU patients, *P *< 0.001). suPAR serum concentrations did not correlate with age or sex, either in controls or in patients (data not shown).

Among the total cohort of ICU patients, we could demonstrate a stepwise increase in suPAR levels from critically ill patients, who did not fulfill SIRS criteria, to patients with SIRS and patients with sepsis (Figure 1b). However, differences among non-sepsis patients (SIRS vs non-SIRS) did not reach statistical significance. In order to investigate the impact of the underlying etiology more precisely, we extended our subgroup analyses. Therefore, the cohort of sepsis patients was subdivided into a pulmonary and a non-pulmonary site of infection and the non-sepsis patients were categorized into liver cirrhosis and others (mostly cardiovascular disorders). By these means we could reveal the highest suPAR serum levels in patients with decompensated liver cirrhosis as compared with other causes of critical illness (Figure 1c, Table 2).

**Figure 2:**
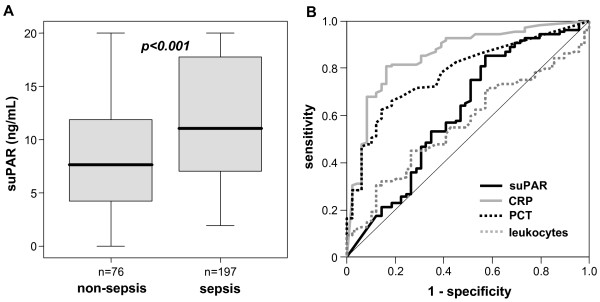
**Serum suPAR concentrations and predictive power for sepsis at ICU admission**. **(A) **In patients with sepsis suPAR serum concentrations were significantly (*P *< 0.001, U-test) higher as compared with patients with non-septic etiology of critical illness. **(B) **Receiver operating characteristic (ROC) curve analyses comparing the diagnostic power in predicting sepsis of suPAR (black line, area under the curve = AUC 0.615) with classical markers of inflammation and bacterial infection, C-reactive protein (CRP; grey line, AUC 0.857), procalcitonin (PCT; dotted black line, AUC 0.780) and white blood cell count (leukocytes; dotted grey line, AUC 0.564).

We hypothesized that elevated suPAR levels could discriminate between sepsis and non-sepsis critical illness. Indeed, patients with sepsis demonstrated significantly higher suPAR levels in comparison to patients without sepsis (Figure 2a, Table 1). We, therefore, tested whether the predictive value of suPAR was equal or superior to classical markers of inflammation and bacterial infection by using ROC curve analyses comparing suPAR with CRP, procalcitonin (PCT) and white blood cell count. Whereas CRP and PCT achieved AUC statistics of 0.857 and 0.780, suPAR and white blood cell count only reached AUC of 0.615 and 0.564 (Figure 2b). Collectively, our data demonstrated the strong elevation of suPAR in critically ill patients upon admission to the ICU, but suPAR did not show superiority compared to classical biomarkers in predicting sepsis.

### suPAR serum concentrations remain stably elevated during the clinical course within the first week after ICU admission

**Figure 3:**
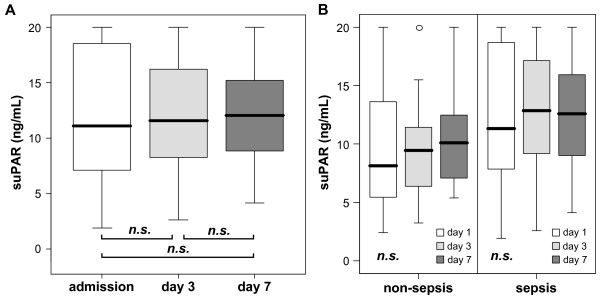
**Sequential measurements of suPAR serum concentrations during the first week of intensive care treatment**. **(A) **Serum suPAR levels were assessed at admission (Day 1), at Day 3 and Day 7 in all critically ill patients. Overall, serum suPAR concentrations did not significantly change during the course of disease within the first week after admission to the ICU (Wilcoxon-Test). **(B) **In subgroup analyses for sepsis and non-sepsis patients as well, no significant changes of suPAR serum levels within the first week of ICU treatment could be detected.

We next investigated whether suPAR levels changed in individual patients during the first week of ICU treatment. Within the first week, the following surviving patients were discharged from the ICU: n = 8 at Day 1, n = 30 between Day 1 and Day 3, and n = 46 between Day 4 and Day 7. On the other hand, n = 3 died during the first 24 hours, n = 18 at days 2 and 3, and n = 16 between Day 4 and Day 7. For patients that were treated at the ICU for at least three or even seven days, we performed longitudinal suPAR measurements at Day 3 and Day 7. There was a tendency towards rising suPAR levels in longitudinal measurements, but serum suPAR concentrations did not significantly change during the course of disease within the first week (paired Wilcoxon-test, not significant). This was found for the total cohort of all critically ill patients as well as for the subgroups of sepsis and non-sepsis patients (Figure 3a,b). These data indicated that the elevation of suPAR levels in ICU patients remain rather stable within the first week of intensive care treatment measures.

### suPAR serum concentrations at admission to the ICU are closely correlated to biomarkers of inflammation, organ function and clinical scores

To determine the factors possibly promoting elevated serum suPAR levels in critically ill patients, correlation analyses with extensive sets of laboratory parameters were performed. For these analyses, serum suPAR levels at admission were applied, in order to exclude possible confounding effects due to patients that died or were discharged from the ICU during the first week. At admission to the ICU, serum suPAR concentrations in the total cohort and the subgroups of sepsis and non-sepsis patients were closely correlated to markers of inflammation and bacterial infection, as TNF-α (r = 0.571, *P *< 0.001), CRP (r = 0.411, *P *< 0.001) and PCT (r = 0.468, *P *< 0.001; Table 3). With regard to organ function we could reveal strong associations with renal and hepatic function for the total study cohort and the subgroups of sepsis and non-sepsis patients. Specifically, we could demonstrate an inverse association with renal function as displayed by highly significant correlations with the glomerular filtration rate of cystatin C (r = -0.649, *P *< 0.001), cystatin C (r = 0.638, *P *< 0.001), creatinine (r = 0.352, *P *< 0.0001) and urea (r = 0.400, *P *< 0.001) serum concentrations (Table 3), indicating renal clearance of suPAR. Interestingly, liver function could be identified as a strong predictor of serum suPAR, as suPAR levels inversely correlated with parameters reflecting the liver's biosynthetic capacity, namely albumin (r = -0.444, *P *< 0.001), pseudocholinesterase activity (r = -0.492, *P *< 0.001), IGF-1 concentrations (r = -0.379, *P *< 0.001) and antithrombin III (ATIII) (r = -0.416, *P *< 0.001). On the other hand, we detected a close direct correlation of suPAR with biomarkers indicating cholestasis, as bilirubin (r = 0.243, *P *< 0.001), gamma-glutamyl-transferase (r = 0.354, r < 0.001) and alkaline phosphatase (r = 0.441, *P *< 0.001). In a multivariate linear regression analysis with suPAR as the dependent variable, TNF-α (*P *= 0.015), CRP (*P *= 0.038), urea (*P *= 0.06) and pseudocholinesterase (*P *= 0.016) were independent predictors of elevated suPAR (R = 0.703, *P *< 0.001 for this model).

**Table 3:** Correlations with suPAR serum concentrations at admission day

	All patients		Sepsis		Non-sepsis	
Parameters	r	*P*	r	*P*	r	*P*
						
***Markers of inflammation***						
TNF-α	0.571	< 0.001	0.555	< 0.001	0.583	0.001
CRP	0.411	< 0.001	0.408	< 0.001	-	n.s.
Procalcitonin	0.468	< 0.001	0.437	< 0.001	0.418	0.003
						
***Markers of organ function***						
Creatinine	0.352	< 0.001	0.273	< 0.001	0.430	< 0.001
Urea	0.400	< 0.001	0.326	<0.001	0.458	< 0.001
Cystatin C	0.638	< 0.001	0.557	< 0.001	0.724	< 0.001
Cystatin C GFR	-0.649	< 0.001	-0.583	< 0.001	-0.714	< 0.001
Bilirubin	0.243	< 0.001	0.194	0.012	0.413	< 0.001
yGT	0.354	< 0.001	0.350	< 0.001	0.367	0.002
AP	0.441	< 0.001	0.379	< 0.001	0.599	< 0.001
PCHE	-0.492	< 0.001	-0.449	< 0.001	-0.438	< 0.001
Albumin	-0.444	< 0.001	-0.387	< 0.001	-0.430	0.003
INR	0.300	< 0.001	0.214	0.006	0.416	< 0.001
ATIII	-0.416	< 0.001	-0.300	0.006	-0.507	0.002
IGF-1	-0.379	< 0.001	-0.247	0.029	-0.625	< 0.001
Base excess	-0.217	0.001	-0.291	< 0.001	-	n.s.
						
***ICU treatment measures***						
FiO_2_	0.230	0.043	-	n.s.	-	n.s.
PEEP	0.319	0.004	0.265	0.042	-	n.s.
Norephinephrine dose	0.131	0.050	0.202	0.011	-	n.s.
						
***Clinical scoring***						
APACHE II	0.345	< 0.001	0.353	< 0.001	-	n.s.
SOFA	0.337	0.004	-	n.s.	-	n.s.
SAPS2	0.271	0.004	0.346	0.002	-	n.s.

AP, alkaline phosphatase; APACHE, Acute Physiology and Chronic Health Evaluation; ATIII, antithrombin III; CRP, C-reactive protein; FiO_2_, fraction of inspired oxygen; GFR, glomerular filtration rate; IGF-1, insulin-like growth factor 1; INR, international normalized ratio; PCHE, pseudocholinesterase; PEEP, Positive end-expiratory pressure; r, correlation coefficient; *P*, *P*-value; r and p-values by Spearman rank correlation; SAPS, simplified acute physiology score; SOFA, sequential organ failure assessment; TNF-α, tumor necrosis factor α; yGT, gamma-glutamyl-transferase.

For the total cohort of critically ill patients a strong association of suPAR serum concentrations at admission to the ICU and established clinical scores like APACHE II (r = 0.345, *P *< 0.001), SOFA (r = 0.337, *P *= 0.004) and SAPS2 (r = 0.271, *P *= 0.004) could be shown, suggesting that suPAR levels are closely linked to disease severity in critical illness. This result was corroborated by (relatively weaker) correlations between suPAR and ICU treatment measures such as ventilation settings and vasopressor doses (Table 3). However, these correlations cannot be considered significant, if *post-hoc *adjustments (Bonferroni) are applied, as the level of significance would then be *P *< 0.002 (instead of *P *< 0.05).

### suPAR is a strong predictive marker for ICU- and overall survival in critically ill patients

We used Cox regression analyses and Kaplan-Meier curves to assess the impact of suPAR serum concentrations on ICU- and overall survival among all critically ill patients and the subgroups of sepsis and non-sepsis patients over a long-term follow-up period (median observation time 348 days, range 29 to 884).

**Figure 4:**
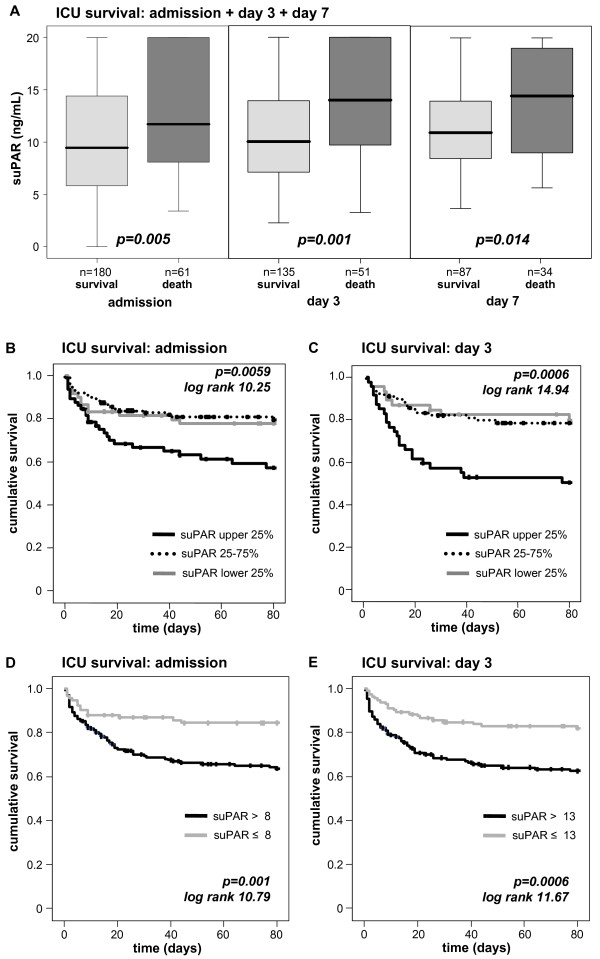
**Prediction of ICU mortality by sequential suPAR serum concentrations**. **(A) **Patients that die during the course of ICU treatment had significantly higher serum suPAR levels on admittance to ICU (*P *= 0.005), on Day 3 (*P *= 0.001) and Day 7 (*P *= 0.014) than survivors. **(B **and **C) **Kaplan-Meier survival curves of ICU patients are displayed, showing that patients with suPAR levels of upper quartile (on admission >15 ng/mL, on Day 3 > 15 ng/mL; black) had an increased short-term mortality at the ICU as compared to patients with suPAR serum concentrations of lower quartile (on admission < 6 ng/ml, on Day 3 < 7 ng/ml; grey) or middle 50% (dotted line). *P*-values are given in the figure. **(D **and **E) **Kaplan-Meier survival curves of ICU patients are displayed, showing that patients with high suPAR levels (on admission > 8 ng/mL, on Day 3 > 13 ng/mL; grey) had an increased short-term mortality at the ICU as compared to patients with low suPAR serum concentrations. *P*-values are given in the figure.

Interestingly, patients that died during the subsequent ICU treatment showed significantly higher suPAR levels at admission as well as on days 3 and 7 (Figure 4a, Table 4). Low suPAR levels upon admission to the ICU, on Day 3 and Day 7 were a strong prognostic predictor for ICU-survival (admission *P *= 0.003, Day 3 *P *< 0.001, Day 7 *P *= 0.013; Cox regression analyses). In multivariate Cox regression analyses, including markers of inflammation/infection (CRP, PCT), hepatic (albumin, international normalized ratio (INR)) and renal (creatinine) function at admission, suPAR remained an independent significant prognostic parameter (hazard ratios and *P*-values are presented in Table 5). Kaplan-Meier curves displayed that patients with suPAR levels of the upper quartile had the highest mortality (Figure 4b,c). We found the best cut-off value to discriminate survivors from non-ICU-survivors for serum suPAR of 8 ng/mL at Day 1 or 13 ng/mL at Day 3 (Figure 4d,e). Of note, suPAR serum concentrations were not found to predict the length of ICU stay (data not shown).

**Table 4:** suPAR serum concentrations and association with survival

	Admission	Day 3	Day 7
Survivor ICU	*n = *180	*n = *135	*n = *87
suPAR median (range)(ng/mL)	9.47 (0 to 20)	10.07 (2.3 to 20)	10.93 (3.6 to 20)
**Death ICU**	n = 61	n = 51	n = 34
suPAR median (range) (ng/mL)	11.73 (3.4 to 20)	14.01 (3.2 to 20)	14.46 (5.6 to 20)
			
**Survivor overall**	n = 116	n = 95	n = 58
suPAR median (range) (ng/mL)	7.98 (2.31 to 20)	9.84 (2.33 to 20)	10.66 (3.67 to 20)
**Death overall**	n = 115	n = 85	n = 60
suPAR median (range) (ng/mL)	11.25 (0 to 20)	12.10 (3.10 to 20)	12.75 (5.38 to 20)

**Table 5:** Multivariate Cox regression analysis for suPAR levels at admission to predict ICU mortality

Parameter	Unadjusted HR (95% CI)	*P-value*	Adjusted HR (95% CI)	*P-value*
C-reactive protein	-	*n.s.*	-	*n.s.*
procalcitonin	-	*n.s.*	-	*n.s.*
albumin	0.940 (0.907 to 0.973)	*< 0.001*	0.926 (0.885 to 0.969)	*0.001*
INR	1.723 (1.302 to 2.28)	*< 0.001*	-	*n.s.*
creatinine	-	*n.s.*	-	*n.s.*
suPAR	1.067 (1.022 to 1.113)	*0.003*	1.089 (1.027 to 1.154)	*0.004*

CI, confidence interval; INR, international normalized ratio; HR, hazard ratio.

**Figure 5:**
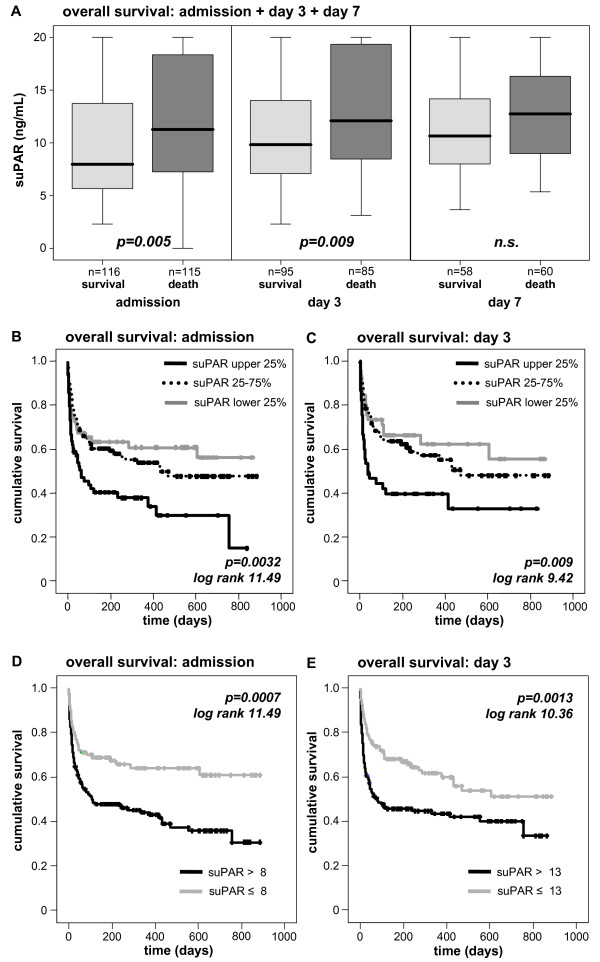
**Prediction of long-term mortality by sequential suPAR serum concentrations**. **(A) **Serum suPAR concentrations were significantly associated with the overall survival of critically ill patients. Survivors had significantly lower serum suPAR levels on admittance to ICU (*P *= 0.005) and on Day 3 (*P *= 0.009). **(B **and **C) **Kaplan-Meier survival curves of ICU patients are displayed, showing that patients with suPAR levels of upper quartile (on admission > 15 ng/mL, on Day 3 > 15 ng/mL; black) had an increased short-term mortality at the ICU as compared to patients with suPAR serum concentrations of lower quartile (on admission < 6 ng/ml, on Day 3 < 7 ng/ml; grey) or middle 50% (dotted line). *P*-values are given in the figure. **(D **and **E) **Kaplan-Meier survival curves of ICU patients are displayed, showing that patients with high suPAR levels (on admission > 8 ng/mL, on Day 3 >13 ng/mL; grey) had an increased overall mortality in the long-term follow-up as compared to patients with low suPAR serum concentrations. *P*-values are given in the figure.

As depicted in Table 4, 27.8% of the patients died at the ICU. However, 50.2% of all patients died overall, meaning that an additional 22.4% from the total cohort died during the follow-up period of approximately one year. Extending our findings from short-term ICU-survival, we could reveal that patients that will die during long-term follow-up had significantly higher suPAR levels than survivors at ICU admission and Day 3 (Figure 5a; Table 4). By Cox regression analyses, high suPAR levels at admission (*P *= 0.001) and Day 3 (*P *= 0.001) predicted long-term mortality in critically ill patients. We also observed a trend for suPAR levels determined at Day 7; however, the Cox analysis did not reach statistical significance for the overall survival (*P *= 0.051). Kaplan-Meier curves displayed that patients with suPAR levels of the upper quartile had highest mortality (Figure 5b,c). We found the best cut-off value to discriminate survivors from non-ICU-survivors for serum suPAR of 8 ng/mL at Day 1 or 13 ng/mL at Day 3 (Figure 5d,e).

To test whether a rise in suPAR serum concentrations from Day 1 to Day 3 is associated with an unfavourable prognosis, we compared the individual difference in suPAR levels between Day 3 and Day 1 of ICU-treatment. Survivors and non-survivors did not display different deltas of suPAR serum concentrations between Day 3 and Day 1 (data not shown).

### suPAR has superior prognostic value as compared with single parameters of inflammation and organ dysfunction in critically ill patients

**Figure 6:**
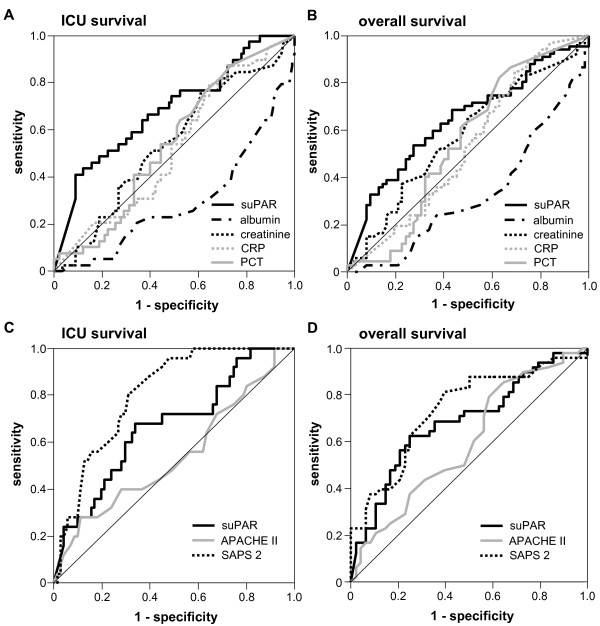
**Prognostic value of suPAR for mortality in comparison with other biomarkers and clinical scores in critical illness**. **(A **and **B) **ROC curve analyses comparing the prognostic value of suPAR at admission for ICU survival/overall survival (black line, AUC 0.684/0.642) with albumin (dashed black line, AUC 0.294/0.329) and creatinine (dotted black line, AUC 0.542/0.576) as makers of hepatic and renal function as well as CRP (grey dotted line, AUC 0.524/0.531) and PCT (grey line, AUC 0.545/0.550) as markers of inflammation and infection. **(C **and **D) **ROC curve analyses comparing the predictive power of suPAR at admission for ICU survival/overall survival (black line, AUC 0.668/0.686) with established clinical ICU scores as APACHE II (grey line, AUC 0.541/0.598) and SAPS 2 (dotted black line, AUC 0.807/0.736).

We used ROC analyses to compare the prognostic value of suPAR at admission for ICU- and overall survival with solitary biomarkers of organ function and inflammation. Albumin and creatinine (at admission) as classical biomarkers for hepatic and renal function achieved AUC statistics for ICU-/overall survival of 0.294/0.329 and 0.542/0.576, respectively. As markers for inflammation and bacterial infection in clinical routine, CRP and PCT reached AUC statistics of 0.524/0.531 and 0.545/0.550. In comparison with these biomarkers of organ dysfunction and inflammation, suPAR displayed a superior predictive accuracy for both ICU- and overall survival in critically ill patients with an AUC of 0.684/0.642 (Figure 6a,b). However, this predictive power was not superior to SAPS2 (AUC 0.807/0.736), but to APACHE II (AUC 0.541/0.598), as established multi-parameter ICU scores (Figure 6c,d).

## Discussion

In the present study we demonstrate the prognostic impact of suPAR in a large, well characterized cohort of critically ill patients in a medical ICU. We measured suPAR serum concentrations upon admission, prior to intensive care treatment, and during the first week of clinical course. Compared to healthy volunteers critical care patients showed elevated suPAR serum concentrations. Levels were higher in sepsis than in non-sepsis patients. Further subgroup analyses found highest concentrations of suPAR in patients with decompensated liver cirrhosis. In contrast to previous findings in healthy subjects [8], we could not reveal a significant correlation of suPAR either with age or with sex in our cohort of critically ill patients as well as in our control group.

The differences in suPAR levels between sepsis and non-sepsis patients prompted us to test the diagnostic power of suPAR to diagnose sepsis. In a prior study on patients with SIRS and suspicion of having community-acquired infections in a non-intensive care setting suPAR was found to have a low accuracy in diagnosing bacterial infection in SIRS patients [17]. The authors also noted, that the diagnostic accuracies of sepsis markers are highly dependent on the setting in which they are tested and that their results could only be applicable to patients not requiring ICU treatment. To investigate the diagnostic precision of suPAR and classic clinical inflammatory parameter in an intensive care environment we performed ROC analyses. The obtained AUC of suPAR for predicting sepsis in critically ill patients at admission to the ICU was low compared with CRP and PCT.

In contrast to many pro-inflammatory cytokines, suPAR as a potential biomarker exhibits favourable properties due its high stability in serum samples and limited circadian changes in plasma concentrations [6,18,19]. In clinical trials it could be demonstrated that effective treatment of infectious diseases and cancer resulted in a proportional decrease in suPAR serum concentrations, leading to normalization of suPAR levels after full recovery [10,20,21]. Though, it has been unclear whether the decrease of suPAR levels was due to anti-inflammatory treatment and the decline in suPAR serum concentrations had specific impact on prognosis. To verify these observations in critically ill patients, we measured suPAR levels upon admission and during the clinical course within the first week. Serum suPAR concentrations did not significantly differ within the first week, either for the total cohort of critically ill patients or the subgroups of sepsis/non sepsis patients and survivors/non-survivors. The continuous elevation of suPAR underscores the easy applicability in clinical practise during the first week in ICU patients. However, our analysis did not include follow-up measurements of suPAR after full recovery and discharge from the hospital.

suPAR seems to exert a variety of functions in diverse physiological pathways, including the plasminogen activating pathway, inflammation, modulation of cell adhesion, migration and proliferation [10,22-24]. However, It is important to note that suPAR consists of three domains (D1, D2, D3), and proteolytic cleavage between the domains generates different soluble forms of suPAR. The commercial suPARnostic assay (used in this study) measures both full length and D2D3 forms of the highly glucosylated suPAR molecule. It is diffcult to measure suPAR exactly because of the differential glucosylation (suPAR appears as a smear on Western blots) and the mixture of D1D2D3 and D2D3. In a previous study on different forms of suPAR and prognosis in HIV infected patients, the combined clinical value of suPAR D1D2D3 and suPAR D2D3 ('bulk suPAR') was compared to the individual suPAR forms, and the combined measurement (suPAR, expressed in ng/ml) had the strongest prognostic value [25]. Thus, our study allows us to compare the obtained suPAR values to previous studies and to assess the diagnostic/prognostic value of total suPAR levels from a clinical point, but we cannot distinguish between different suPAR cleavage products. We can, therefore, not elucidate the exact pathogenic involvement of the different suPAR forms during critical illness or sepsis.

Importantly, the association of suPAR with inflammation (elevated levels in sepsis patients, independent association with inflammatory cytokines in multivariate linear regression analysis) does not necessarily mean that suPAR exerts proinflammatory actions. A recent study demonstrated that uPA-uPAR interactions are required to control and reduce fibrin-mediated inflammation (in the liver especially) [26]. This suggests that high suPAR levels may not be harmful *per se*, but may serve to rescue and dampen inflammation induced by fibrinogen.

Supporting previous findings in healthy subjects and patients with prostate cancer [6], suPAR serum concentrations were found to be inversely correlated to renal function, indicating renal excretion in critical disease. Subgroup analyses revealed significantly higher levels of suPAR in ICU patients with decompensated liver cirrhosis. Furthermore, suPAR was inversely correlated to pseudocholinesterase, reflecting hepatic synthesis capacity, and is directly associated with parameters indicating cholestasis. Most likely, this could be due to a biliary secretion of suPAR. Interestingly, these two principal associations of elevated suPAR with inflammation and organ function were also identified by a multivariate linear regression analysis revealing that organ function (kidney/urea, liver/pseudocholinesterase) and inflammation (TNFa, CRP) were independent predictors of elevated suPAR.

As serum suPAR has been related to different conditions of immune activation and has been proven as a reliable marker for the level of inflammation, it has been consequentially evaluated as prognostic marker. Moreover, uPAR is highly expressed by the endothelium and various triggers, which are present in sepsis, stimulate release of endothelial uPAR and, hence, increase suPAR levels [26]. It is, therefore, possible that high suPAR concentrations primarily reflect endothelial dysfunction that is a key driver in sepsis morbidity and mortality [27]. In previous studies increased suPAR serum concentrations have been associated with an unfavourable prognosis in individuals with disease due to viral [24,28], bacterial [10,29] or parasitic infections as well as cancer [30] and rheumatic arthritis [31]. Lately, elevated suPAR serum concentrations have been associated with a shortened life expectancy in a general population due to increased risk of developing cancer, cardiovascular disease and diabetes mellitus type 2, proposing suPAR as an early warning biomarker for screening purposes [32]. Most recently, a clinical study matched the prognostic value of easily obtainable urine suPAR with that of plasma suPAR, demonstrating inferior prognostic significance of urine suPAR as compared with plasma suPAR [33].

For the first time we now demonstrated the prognostic impact of suPAR serum concentrations at admittance and during the initial clinical course in a large cohort of critically ill patients in a medical ICU. In fact, critically ill patients with low endogenous suPAR levels upon admission, on Day 3 and on Day 7 had a significantly better outcome (short-term at ICU and long-term during follow-up). Interestingly, in a study investigating HIV-positive patients with low CD4 count (<200 cells/μl), high suPAR levels were significantly associated with increased mortality [34]. The authors suggested the initiation of antiviral therapy in patients with low CD4 count and suPAR serum concentration at a maximum of 6 ng/mL, a cut-off for a high risk situation, which is similar to the cut-off values identified by our study.

Comparing suPAR with single, routinely used clinical biomarkers of organ dysfunction and inflammation, suPAR displayed better predictive accuracy for both ICU and overall survival in critically ill patients. Regarding established clinical scoring systems, the SAPS2 score revealed the best predictive accuracy for survival as compared with suPAR and APACHE II score. Given the high prognostic value of suPAR revealed by our study, future studies should address the implementation of suPAR measurements to improve the power of established scoring systems in large cohorts of critically ill patients.

Based on our findings that serum suPAR is a strong and robust marker of mortality risk, one could speculate that suPAR eventually may help to triage patients at the Emergency Room for relocation to the ICU or to guide therapeutic decisions within the first week of ICU treatment, especially in "high suPAR" patients with a high likelihood of death. Future studies are needed to test this hypothesis and also to assess whether the response to efficient therapy will correlate with a decrease in suPAR and whether this in turn is associated with a better chance of survival.

## Conclusions

Our study identifies suPAR as a stable and robust marker in critical ill patients to assess disease severity and mortality risk. Low individual suPAR levels are highly predictive for ICU- and overall survival. In line with prognostic properties, suPAR is closely correlated to inflammation, and renal and hepatic dysfunction, which are central pathophysiological and therapeutic targets in critical disease. At present, it is unclear whether serum suPAR is truly causatively involved in mechanisms of critical disease resulting in high mortality or whether it reflects general inflammation in critical illness. Further studies are required for a satisfying understanding of the biochemical properties and regulatory mechanisms of suPAR in critical disease in order to evaluate whether suPAR could also be a potential novel therapeutic target in critically ill patients.

## Key messages

• Soluble urokinase plasminogen activator receptor (suPAR) is derived from various immunologically active cells and suggested to reflect general inflammation and the extent of resulting immune-activation.

• In critically ill patients suPAR serum concentrations are significantly increased compared to healthy controls, especially in patients with sepsis, and remain stably elevated within the first week of critical disease.

• suPAR is closely correlated to inflammatory markers indicating substantial involvement in systemic inflammatory response in critical disease.

• suPAR correlates to renal and liver function indicating possible routes of clearance.

• suPAR is a strong predictor for ICU and overall survival in critically ill patients.

## Abbreviations

APACHE II: Acute Physiology and Chronic Health Evaluation II; AP: alkaline phosphatase; ATIII: antithrombin III; AUC: area under the curve; BMI: body mass index; CRP: C-reactive protein; ELISA: enzyme-linked immunosorbent assay; yGT: gamma-glutamyl-transferase; GFR: glomerular filtration rate; ICU: intensive care unit; IGF-1: insulin-like growth factor 1; IL-6: interleukin 6; IL-10: interleukin 10; INR: international normalized ratio; *P: P*-value; PCHE: pseudocholinesterase; r, correlation coefficient; ROC: receiver operating characteristic; SAPS: Simplified Acute Physiology Score; SIRS: systemic inflammatory response syndrome; SOFA: Sequential Organ Failure Assessment; suPAR: soluble urokinase plasminogen activator receptor; TNF-α: tumor necrosis factor α; uPA: urokinase plasminogen activator; uPAR: urokinase plasminogen activator receptor

## Competing interests

The authors declare that they have no competing interests.

## Authors' contributions

AK, FT and CT designed the study, analyzed data and wrote the manuscript. SV performed the suPAR measurements. CK assisted with statistical analyses. EY contributed further laboratory measurements, and ES, HD, HZ, and AH collected data and assisted in patient recruitment.
